# *Drosophila *selenophosphate synthetase 1 regulates vitamin B6 metabolism: prediction and confirmation

**DOI:** 10.1186/1471-2164-12-426

**Published:** 2011-08-24

**Authors:** Kwang Hee Lee, Myoung Sup Shim, Jin Young Kim, Hee Kyoung Jung, Eunji Lee, Bradley A Carlson, Xue-Ming Xu, Jin Mo Park, Dolph L Hatfield, Taesung Park, Byeong Jae Lee

**Affiliations:** 1 Department of Biological Sciences, Seoul National University, Seoul 151-742, Korea; 2 Interdisciplinary Program in Bioinformatics, Seoul National University, Seoul 151-742, Korea; 3 Department of Statistics, Seoul National University, Seoul 151-742, Korea; 4 Laboratory of Cancer Prevention, Center for Cancer Research, National Cancer Institute, National Institutes of Health, Bethesda, MD 20892, USA; 5 Department of Dermatology, Massachusetts General Hospital and Harvard Medical School, Boston, MA, 02114, USA

## Abstract

**Background:**

There are two selenophosphate synthetases (SPSs) in higher eukaryotes, SPS1 and SPS2. Of these two isotypes, only SPS2 catalyzes selenophosphate synthesis. Although SPS1 does not contain selenophosphate synthesis activity, it was found to be essential for cell growth and embryogenesis in *Drosophila*. The function of SPS1, however, has not been elucidated.

**Results:**

Differentially expressed genes in *Drosophila *SL2 cells were identified using two-way analysis of variance methods and clustered according to their temporal expression pattern. Gene ontology analysis was performed against differentially expressed genes and gene ontology terms related to vitamin B6 biosynthesis were found to be significantly affected at the early stage at which megamitochondria were not formed (day 3) after *SPS1 *knockdown. Interestingly, genes related to defense and amino acid metabolism were affected at a later stage (day 5) following knockdown. Levels of pyridoxal phosphate, an active form of vitamin B6, were decreased by *SPS1 *knockdown. Treatment of SL2 cells with an inhibitor of pyridoxal phosphate synthesis resulted in both a similar pattern of expression as that found by *SPS1 *knockdown and the formation of megamitochondria, the major phenotypic change observed by *SPS1 *knockdown.

**Conclusions:**

These results indicate that SPS1 regulates vitamin B6 synthesis, which in turn impacts various cellular systems such as amino acid metabolism, defense and other important metabolic activities.

## Background

Selenium has been reported to provide many health benefits in animals, including humans, when obtained from the diet in adequate amounts. For example, selenium has been known to play roles in cancer prevention, aging retardation, immune augmentation, prevention of heart diseases, muscle development and development [[1-4] and references therein]. Many of the health benefits of selenium are mediated by selenoproteins, which contain selenocysteine (Sec) as a selenium containing amino acid [3].

Selenophosphate synthetase (SPS) synthesizes selenophosphate (SeP), the active selenium donor in Sec biosynthesis, using selenide and ATP as substrates [5]. SeP serves as a selenium donor during Sec biosynthesis [6]. Sec is contained in all selenoproteins [7]. SPS was first isolated from *Escherichia coli *as one of the enzymes involved in selenoprotein synthesis and was designated SelD [8]. Only one type of SPS, SelD, exists in lower eukaryotes and eubacteria, however, there are two isoforms of SPS, SPS1 and SPS2, that occur in higher eukaryotes [9]. One of the major differences in the sequences between SPS1 and SPS2 is that SPS1 has an arginine at the position corresponding to Sec in SPS2 [10].

Although it is not clear why there are two SPSs in higher eukaryotes, recent studies have shown that SPS2 synthesizes SeP from selenide and ATP *in vitro*, while SPS1 does not have this activity [11]. Loss of function in NIH3T3 cells using RNA interference technology showed that SPS2 is required for selenoprotein biosynthesis, while SPS1 does not affect the biosynthesis of this protein class [12]. While some insects such as the red beetle and silkworm have lost the selenoprotein synthesizing machinery including SPS2, SPS1 is still encoded in the genome of these insects, suggesting SPS1 is required for a function other than SeP synthesis [13].

Although SPS1 does not catalyze SeP biosynthesis, it plays essential roles in the cell. When the gene encoding SPS1 (*SPS1*, also designated *patufet*) was deleted in *Drosophila*, the embryo showed lethality during development [14], and reactive oxygen species (ROS) levels increased [15]. The haploinsufficiency of genes involved in the *Ras*-regulated signaling pathway was also suppressed by *SPS1 *knockout in *Drosophila *[16]. From the finding that the SelD (*E. coli *SPS) mutant of *E. coli *can be complemented by human SPS1 only when L-Sec is supplemented in the medium, it was suggested that SPS1 is involved in the recycling of Sec [4]. However, the means by which SPS1 may be involved in Sec recycling has not been determined. Recently, it was found that the targeted depletion of *SPS1 *by RNA interference in *Drosophila *SL2 cells causes growth inhibition, ROS induction and megamitochondrial formation by increasing intracellular glutamine levels [17]. Interestingly, human SPS1 was found to interact with the soluble liver antigen, which was recently identified as eukaryotic Sec synthase (SecS), and the binding reaction was enhanced by Sec tRNA methylase designated SECp43 [18,19]. It should be noted that SecS is a pyridoxal phosphate (PLP)-dependent enzyme and, therefore, the uptake and/or activation of vitamin B6 may be related to selenium metabolism [20,21].

Vitamin B6 is a water-soluble compound that contains a pyridine ring. Vitamin B6 is present in nature as several different forms such as pyridoxal (PL), pyridoxine (PN), pyridoxamine (PM) and their 5'-phosphorylated forms [22]. Before use, these vitamers are converted to PLP, which is the metabolically active form. PLP is used as a cofactor for PLP-dependent enzymes, where the pyridine ring acts as an electron sink during enzymatic reactions. Since animals, including humans, cannot synthesize vitamin B6, they must obtain it from their diet [23]. PLP can be synthesized through several different pathways, and two types of enzymes, kinases and oxidases, participate in these pathways. For PM to be converted to PLP, it is first phosphorylated by a kinase (PL/PM/PN kinase) to form pyridoxamine phosphate (PMP), and then the PMP is oxidized to form PLP using an oxidase (PMP/PNP oxidase). PN can also be converted to PLP using the same kinase and oxidase used for PM. In this case, the phosphorylated intermediate is pyridoxine phosphate (PNP). However, PL can be directly converted to PLP by phosphorylation using a kinase [24]. Therefore, kinases and oxidases are important enzymes for PLP synthesis.

There are more than 100 PLP-dependent enzymes in a cell that perform essential roles in various metabolic pathways including amino acid metabolism (such as amino acid synthesis and degradation), fatty acid metabolism (such as the synthesis of polyunsaturated fatty acids), and carbohydrate metabolism (such as the breakdown of glycogen) [[23] and references therein]. The PLP-dependent enzymes that participate in amino acid metabolism can be classified into 4 categories: transaminase, racemase, decarboxylase and α,β-eliminase [25]. Interestingly, the biosynthesis of Sec can be mediated by cystathionine β-synthase (CBS) using serine as a precursor and it can also be synthesized by cystathionine γ-lyase (CGL) from selenocystathionine [26,27]. Both CBS and CGL are PLP-dependent enzymes [28]. In addition, enzymes that are involved in the degradation of Sec, such as selenocysteine lyase (SCL), D-selenocystine α, and β-lyase, use PLP as a cofactor [29]. Recently, it was found that SCL can interact with SPS1 [30]. Therefore, it seems that vitamin B6 participates in the metabolism of Sec, i.e., in the biosynthesis and/or decomposition of Sec.

In the present study, we found that the knockdown of *SPS1 *led to the down regulation of genes involved in PLP biosynthesis, which, in turn, induced the formation of megamitochondria and the expression of genes responsible for innate immunity. Our findings suggest that SPS1 primarily regulates PLP biosynthesis, and the intracellular PLP level affects various biological processes such as amino acid metabolism, megamitochondrial formation and innate immune response.

## Results

### Identification and temporal clustering of differentially expressed genes

**Figure 1:**
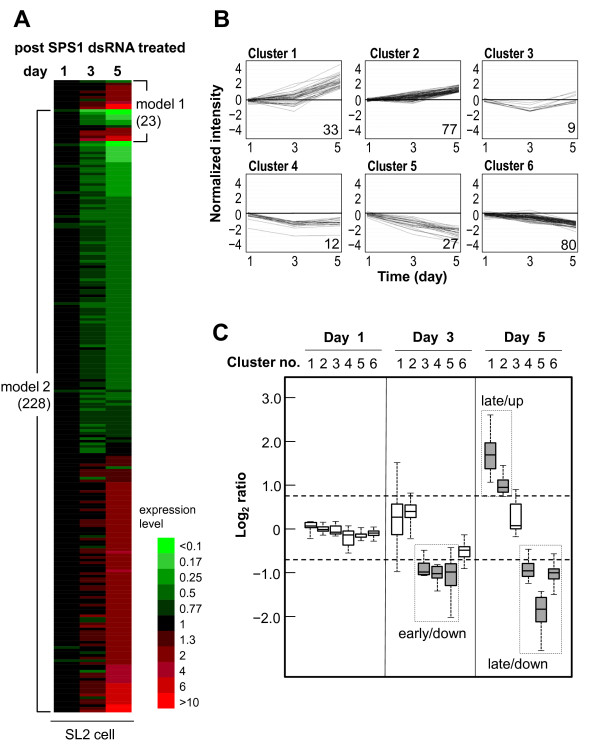
**Identification and clustering of DEGs**. **A**. DEGs expression profiles ordered by fold changes on day 5 in each model. The number in bracket represents the gene numbers including each model. Genes selected by both model (model 1 and 2) overlapped between model 1 and model 2. **B**. DEGs were classified into six clusters according to their temporal expression patterns using SOM clustering methods. The number in each panel represents the number of genes in each cluster. Normalized intensities are log_2_-values of signal intensity. **C**. The range of expression ratios of DEGs in each cluster was drawn with a box plot. The line in each box designates the median quartile (Q2). Dashed lines designate the threshold values (log_2 _ratio of +0.75 and -0.75) for determining clusters of genes whose expressions were changed significantly. The dotted boxes represent the clusters showing their inter-quartile ranges (IQRs) and are the outliers of the threshold, and the genes in those clusters were selected as gene sets for GO analysis.

After the addition of double stranded RNAs targeting SPS1 to the culture medium, total RNAs were isolated on days 1, 3 and 5 after treatment and subjected to microarray analysis using Affymetrix microchips (GEO accession number: GSE 17685). Because megamitochondrial formation begins 3 days after knockdown [17], transcriptomes were analyzed before and after megamitochondrial formation to find the primary target of SPS1. The knockdown efficiency was approximately 90% which was similar with that obtained in the previous work [17]. The log_2 _values of signal intensity of 18,952 transcripts on each chip were obtained after normalization. By performing two-way ANOVA analysis (adjusted P-value < 0.1) against the log_2 _values of signal intensities of transcripts, a total of 238 genes were found to be different in their expression between knockdown and control cells. Twenty-three genes were selected by model 1 and 227 genes by model 2, with 12 genes being common between the two models (Figure 1A; Additional File 1 shows the list of DEGs).

To analyze the expression pattern of DEGs generated by *SPS1 *knockdown, clustering of DEGs was performed according to their temporal expression using by self-organizing map (SOM) algorithms [31]. As a result, the DEGs were classified into 6 clusters (Figure 1B). Genes belonging to cluster 1 (33 genes) showed continuous increase in their expression by *SPS1 *knockdown, and most of them showed more than 4-fold increase on day 5. The expression patterns of genes in cluster 2 (77 genes) were similar to those of cluster 1, but the average expression level was lower than that of cluster 1. Genes in cluster 3 (9 genes) showed down-up patterns of expression. The expression of cluster 4 genes (12 genes) was decreased until day 3, and the expression level was maintained afterward. The expression pattern of genes in cluster 5 (27 genes) was a down-down type. Genes in cluster 6 (80 genes) showed an expression pattern similar to that of cluster 5 genes. However, the average level of expression of cluster 5 genes was much lower than that of cluster 6 genes.

Using six clusters resulted from above, the expression ratios of DEGs composing a cluster were drawn as a box plot according to their sampling date (days 1, 3, and 5). As shown in Figure 1C, the median values (Q2s) of all clusters were close to zero on day 1. However, Q2s of clusters 3, 4 and 5 on day 3 were significantly decreased. On day 5, Q2s of clusters 1 and 2 were significantly increased, while those of clusters 4, 5 and 6 decreased. The interquartile ranges (IQRs) of each cluster were compared to select cluster(s) whose IQRs were significantly deviated. Clusters 3, 4 and 5 revealed significant down regulation compared to the other clusters on day 3. The IQRs of those clusters on day 3 were lower than -0.75. Therefore, the threshold to select clusters whose expression was significantly changed at a specific sampling date was set to the absolute value of 0.75 (see the dashed lines in Figure 1C). A gene pool composing the selected clusters that showed the same expression pattern at the same sampling date was used as a gene-set for gene ontology analysis. As shown in Figure 1C, there is no cluster showing that their IQRs were located at the outside of the threshold range (-0.75~ +0.75) on day 1; thus, no gene was selected for GO analysis from day 1 samples. However, on day 3, the IQRs of clusters 3, 4 and 5 were lower than the lower threshold (-0.75), and the genes in these clusters were defined as the early/down gene-set because their expressions were decreased. Clusters 1 and 2 showed a significant increase in their expression on day 5, and the genes in those clusters were defined as the late/up gene-set. On the other hand, genes in clusters 4, 5 and 6 showed significant down-regulation in their expression, and they were defined as the late/down gene-set (the dotted boxes in Figure 1C; Additional File 2 for the list of genes in these gene-sets).

### Identification of statistically overrepresented biological processes by gene ontology analysis

To predict overrepresented metabolic pathway or biological process that is significantly affected by *SPS1 *knockdown, gene ontology (GO) analysis [32] was performed with 3 gene-sets (early/down, late/up and late/down) previously defined using BinGO software [33]. The parameters for statistical test and multiple testing correction were used to binomical test and Bonferroni family-wise error rate (FWER) correction [34], respectively. As a result, total 29 GO biological process terms and 23 genes, which are included in each GO term, were selected. (Table 1; see also Additional File 3). The terms related to vitamin B6 biosynthesis were selected as significant GO terms from the early/down gene-set (p-value = 2.48 e-02). Changing the parameters for statistical tests and multiple testing corrections to Benjamini-Hochberg false discovery rate (FDR) [34] and hypergeometric test did not change the results (Additional File 4), suggesting vitamin B6 biosynthesis is the only significant biological process affected by *SPS1 *knockdown at the early stage. GO terms selected from the late/up gene-set could be categorized into two distinct biological processes: defense (immune) response and carboxylic acid (amino acid) metabolism (Table 1). Both defense response (p = 6.22 e-08) and carboxylic acid processes (p = 8.17 e-05) were selected with significantly high probabilities. Interestingly, 15 genes among 21 genes (72%) selected from the late/up gene-set are known to participate in defense response. In addition, 7 of 15 defense response genes encode antimicrobial peptide (AMP). No GO term was selected from the late/down gene-set. These results strongly suggest that SPS1 affects vitamin B6 biosynthesis at the early stage and then defense response and amino acid metabolism through vitamin B6 activity.

**Table 1:** List of biological processes selected from gene ontology analysis

Gene-set	Represented biological process	Max. corrected p-value	Selected genes
Early/down	Vitamin B6 biosynthesis	2.48 e-02	*CG11899, CG31472*
Late/up	Defense response	6.22 e-08	*AttB, AttD, CecB, DptB, Dro, Drs, Mtk, egr, pirk, PGRP-LF, PGRP-SD, W, Cyp6a8, Cyp12a4, Toll-7*
	Carboxylic acid metabolism (Amino acid metabolism)	8.17 e-05	*arg, CG8745, Gs1, Oat, Pepck, yellow-f*

### Validation of expression by quantitative PCR

**Figure 2:**
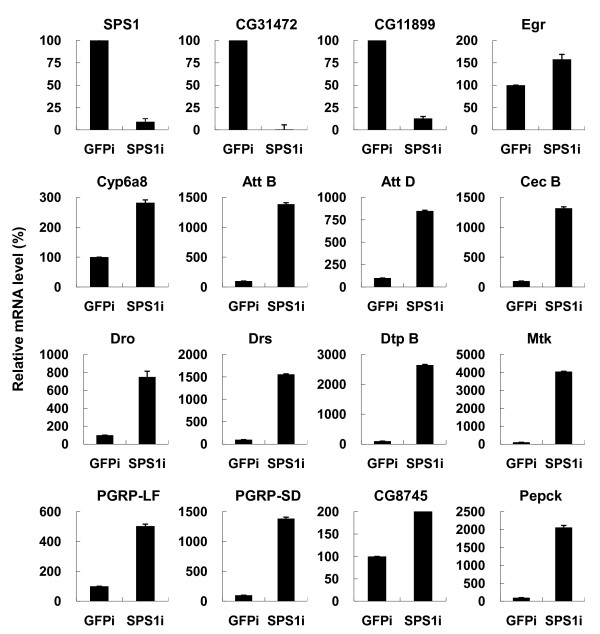
**Validation of the selected genes by quantitative PCR**. Five days after adding dsRNA, the mRNA levels of selected genes were measured by real time RT-PCR using rp49 for normalization. The y axis represents the relative mRNA level of each gene in the cells treated SPS1 dsRNA (SPS1i) to that treated GFP dsRNA (GFPi). The mRNA level of GFPi was set to 100%. The gene symbol is marked above each graph.

Since the cells that were not transfected with double stranded RNA (dsRNA) as control for microarray analysis, it was necessary to confirm that the selected DEGs have the same expression pattern with the cells transfected with control dsRNA. We used GFP dsRNA as a control RNA and quantitative PCR (qPCR) was carried out to measure the expression levels. Of 23 DEGs from the selected GO terms (genes in Table 1), 15 genes were arbitrarily chosen, and their expressions were compared between *SPS1 *knockdown and GFP dsRNA treated control cells. As shown in Figure 2, all tested genes showed the same pattern of expression as that obtained from microarrays. It should be addressed that all the genes involved in vitamin B6 synthesis and encoding AMP were tested and showed the same expression patterns.

### Intracellular pyridoxal phosphate level was decreased by *SPS1 *knockdown

**Figure 3:**
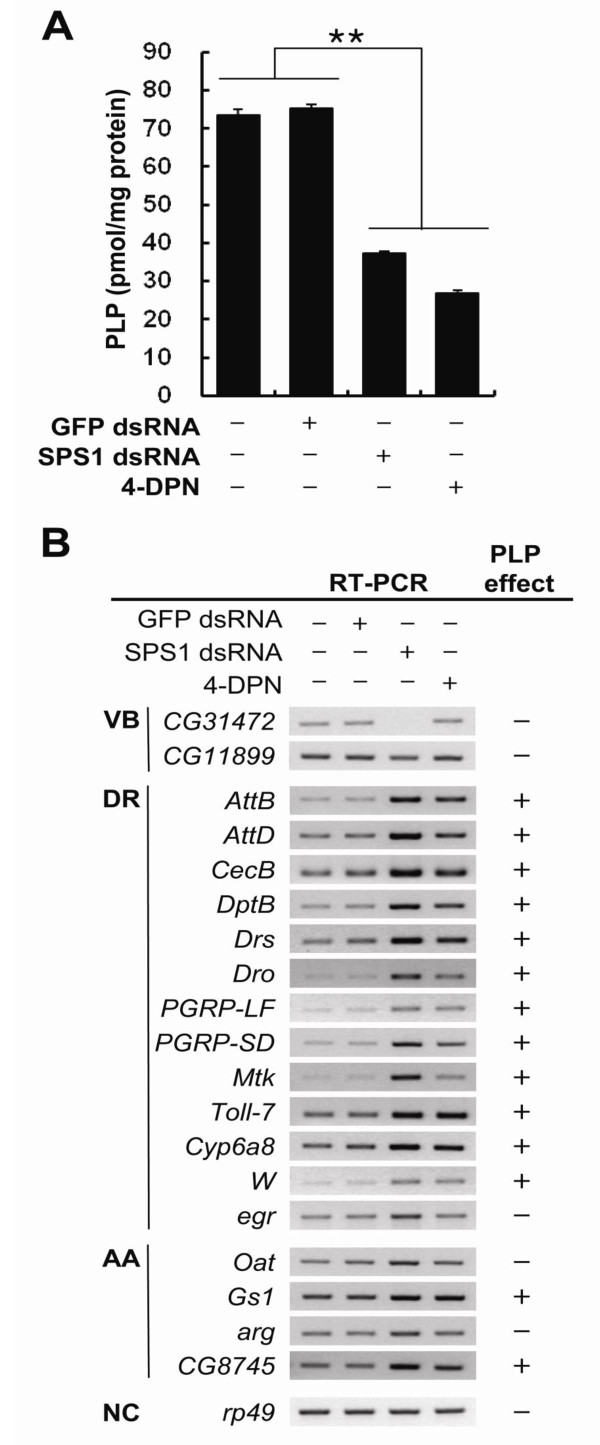
***SPS1 *knockdown causes a decrease in intracellular PLP levels**. **A**. Five days after SPS1 dsRNA or 4-DPN was added to the medium, intracellular PLP levels were measured as described in Methods. dsRNAs and 4-DPN used are shown on the x-axis. Experiments were performed in triplicate and error bars denote the standard deviation from the mean of three independent experiments. Statistical significance was tested by one-way ANOVA followed by Tukey's multiple comparison test. ** indicates significance at p < 0.01. **B**. Five days after treatment of cells with dsRNAs and 4-DPN, expression patterns of genes selected by GO analysis were measured by RT-PCR as described in Methods. Tested genes and GO terms of the gene are shown on the left of each panel. VB, vitamin B6 biosynthesis; DR, defense response; AA, amino acid metabolism; Con, internal control. The effect of 4-DPN on gene expression is represented as the PLP effect. The + and - symbol designate consistency and inconsistency of expression pattern of each gene between *SPS1 *knockdown and 4-DPN treated cells, respectively. rp49 was used as an internal control.

Because GO analysis predicted that vitamin B6 biosynthesis was the only pathway affected at early stage by *SPS1 *knockdown and the expression patterns of genes involved in vitamin B6 synthesis were confirmed, it can be speculated that levels of PLP will decrease by *SPS1 *knockdown. To test this hypothesis, intracellular PLP levels were measured after *SPS1 *knockdown. As shown in Figure 3A, PLP levels in the cells where *SPS1 *was knocked down decreased by approximately twofold compared to the control cells. The PLP concentration in *SPS1 *knockdown cells was 37.23 ± 0.66 pmol/mg protein. On the other hand, the PLP levels in the non-treated control and in GFP dsRNA treated cells (negative control cells) were 73.59 ± 1.31 and 75.37 ± 0.89 pmol/mg protein, respectively. PLP levels in *SPS1 *knockdown cells were similar to those observed in 4-deoxypyridoxine (4-DPN), which is an inhibitor of PLP biosynthesis, treated cells (positive control cells). These results indicate that the function of SPS1 is to regulate the biosynthesis of PLP in the cells.

### Inhibition of PLP biosynthesis and *SPS1 *knockdown showed similar expression patterns

Because intracellular PLP levels were significantly reduced after *SPS1 *knockdown, it can be assumed that PLP biosynthesis is the primary target of SPS1, and the inhibition of PLP synthesis by treating cells with inhibitors will cause similar gene expression patterns as those resulting from *SPS1 *knockdown. To test this hypothesis, *Drosophila *cells were treated with 4-DPN for 5 days, and the expression level of genes selected by GO analysis was measured with RT-PCR. As shown in Figure 3B, the level of expression of the early/down genes (*CG31472 *and *CG11899*) was not changed by 4-DPN treatment. Because 4-DPN inhibits only the function of proteins that participate in PLP synthesis and does not affect the expression of genes encoding those proteins, it is reasonable that 4-DPN does not affect the expression of *CG31472 *and *CG11899*. However, the treatment of 4-DPN affected the expression of genes comprising the late/up and late/down gene-sets. Of the 17 genes tested, 14 genes (82%) showed expression patterns similar to those observed by microarray analysis. It should be noted that the late gene-sets include genes responsible for defense response and amino acid metabolism. These results strongly suggest that PLP synthesis is the primary target of SPS1 and that intracellular PLP levels regulate other important biological processes such as defense system and amino acid metabolism.

### The reduction of intracellular PLP level inhibits cell growth and induces megamitochondrial formation

**Figure 4:**
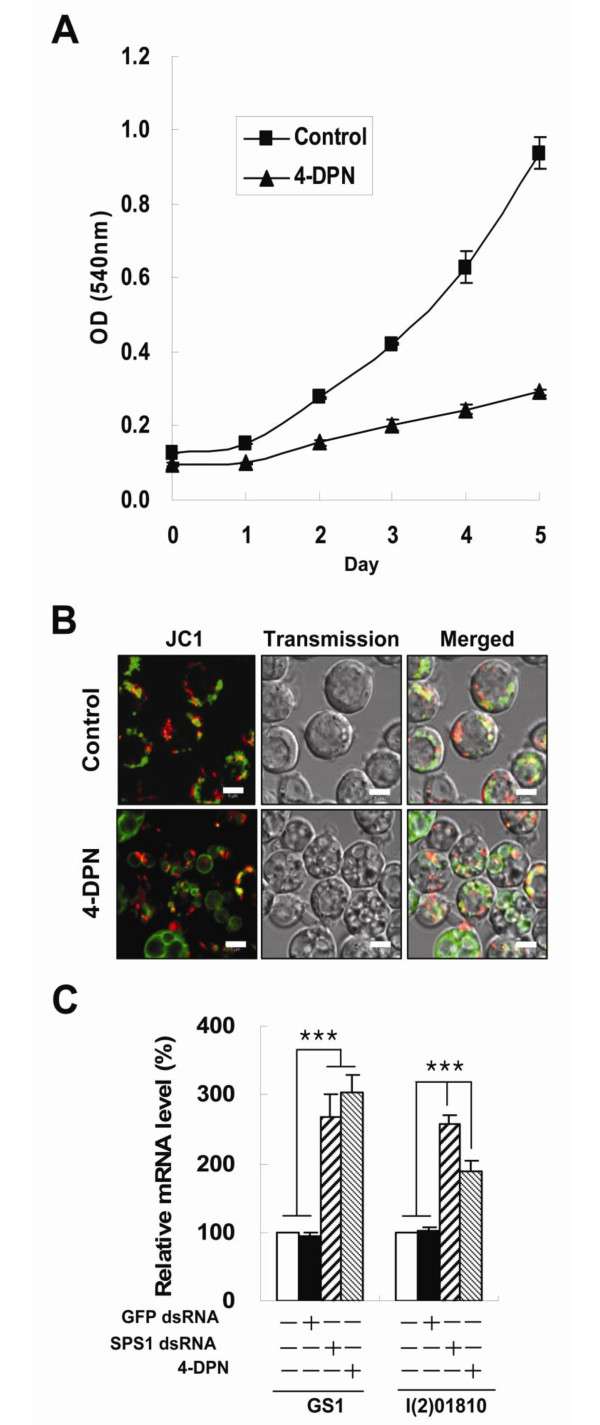
**The effect of PLP synthesis inhibition on cell growth and megamitochondrial formation**. **A**. SL2 cells were seeded in a 96- well plate (2.5 × 10^4 ^cells/well) and the growth rate was examined by the MTT assay in 4-DPN-treated cells as described [17]. Control cells were not treated with 4-DPN. Experiments were performed in triplicate, and error bars denote the standard deviation from the mean of three independent experiments. B. Three days after 4-DPN treatment, cells were stained with JC-1 and then observed under a confocal microscope as described in Methods. Control cells were grown in the absence of 4-DPN. Scale bars represent 5 μm. **C**. Five days after treatment of cells with dsRNAs and 4-DPN, mRNA levels of GS1 and l(2)01810 were measured by realtime RT-PCR as described in Materials and Methods. dsRNAs and 4-DPN treated are shown on the X axis. Experiments were performed in triplicate, and error bars denote the standard deviation from the mean of three independent experiments. Statistical significance was tested by one-way ANOVA followed by Tukey's multiple comparison test. *** indicates significance at p < 0.001.

In our previous study, we discovered that *SPS1 *knockdown leads to cell growth inhibition and induction of megamitochondrial formation [17]. As shown in Figure 4A, cell growth was significantly inhibited after the cells were treated with 4-DPN suggesting that the cell growth retardation induced by SPS1 knockdown was due to vitamin B6 starvation. Another prominent phenotypic change induced by SPS1 knockdown is megamitochondrial formation. *Drosophila *SL2 cells were treated with 4-DPN for 3 days and examined under a confocal microscope after the mitochondria were stained with JC-1. As shown in Figure 4B, the cells treated with 4-DPN formed megamitochondria that were similar to those observed in the *SPS1 *knockdown cells in terms of their size and number. Interestingly, the number of polar mitochondria (red dots in Figure 4B) in 4-DPN treated cells was similar to that in the control cells, and this mitochondrial polarity pattern was also similar to that observed in the *SPS1 *knockdown cells. Since megamitochondria formation can arise from several different pathways, we examined whether megamitochondrial formation occurred by the activation of *Gs1 *and *l(2)01810*. As shown in Figure 4C, both the level of *Gs1 *and *l(2)01810 *expression was increased. These results strongly suggest that the formation of megamitochondria, which is the most prominent phenotype from *SPS1 *knockdown, is induced by the lack of intracellular PLP.

## Discussion

We assumed that the genes whose expression was changed at the early stage after knockdown are involved in the primary target process regulated by SPS1. To identify the primary target, DEGs were isolated after microarray analysis and classified according to their temporal expression pattern; GO terms of early changed DEGs were analyzed using BinGO software. It is interesting that only PLP biosynthesis was predicted from the early/down gene set, even though the parameters were changed. As shown in Table 1, the DEGs in the early/down gene set that are involved in vitamin B6 synthesis are *CG31472 *and *CG11899*. CG31472 is an ortholog of mammalian pyridoxine phosphate oxidase (PNPO), which catalyzes PLP production from PMP and PNP and PL production from PN or PM by oxidizing the substrates [35]. The function of CG11899 was not determined experimentally. However, it has high homology with mammalian phosphoserine aminotransferase and PdxC of *E. coli*, which are responsible for producing 4-phospho-hydroxy threonine, a precursor of the pyridoxine ring [36]. Therefore, it seems that CG11899 plays a role in producing precursors of vitamin B6. Interestingly, intracellular PLP levels were decreased even though only two genes among four genes that are involved in the PLP biosynthesis pathway in *Drosophila *cell were down-regulated (see Additional File 5). This result suggests that these two genes are involved in an essential step of PLP biosynthesis, or SPS1 may also regulate the other proteins involved in PLP biosynthesis post-transcriptionally.

Because PLP is used as a cofactor for various enzymes that are important for many metabolic pathways, including amino acid metabolism, the inhibition of PLP biosynthesis will lead to the inhibition of cell growth. The inhibition of cell growth induced by *SPS1 *knockdown seems to be mediated by a decrease in intracellular PLP levels. Specific inhibition of PLP synthesis by 4-DPN treatment led to growth inhibition (Figure 4A), suggesting the growth inhibition by *SPS1 *knockdown is caused by down-regulation of PLP synthesis.

As described in the Results, down-regulation of genes responsible for PLP synthesis stimulated the expression of DEGs that participate in the defense response. In addition, most of the late gene-sets showed the same pattern of expression as that seen when cells were treated with 4-DPN (Figure 3B). The relationship between vitamin B6 and cellular defense, however, has not been demonstrated before this study. Previously, it was reported that the knockdown of *SPS1 *induced diphthericin expression in *Drosophila *SL2 cell when a genome-wide knockdown was performed [37]. The inhibition of PLP synthesis also induced the expression of various AMPs, including dipththericin. Therefore, SPS1 plays a key role in innate immune responses, including AMP production, by regulating PLP level in the cell. The mechanism by which vitamin B6 regulates the innate immune system remains to be elucidated.

The fact that the treatment of 4-DPN, like *SPS1 *knockdown, induced megamitochondrial formation indicates that intracellular glutamine levels increased with the inhibition of PLP synthesis. Because PLP is used as a cofactor for enzymes that have transaminase activity, it is reasonable to assume that low levels of PLP will lead to the inhibition of synthesis of amino acids such as glutamate or glutamine. However, the inhibition of PLP biosynthesis induced the expression of *Gs1 *and *l(2)01810 *(Figure 4C). These two genes are involved in the increase of intracellular glutamine levels [17]. These results suggest that the lack of PLP in the cell provides a signal for compensatory induction of some genes responsible for amino acid metabolism. PLP regulation of the expression of *Gs1 *and *l(2)01810 *has not been elucidated.

**Figure 5:**
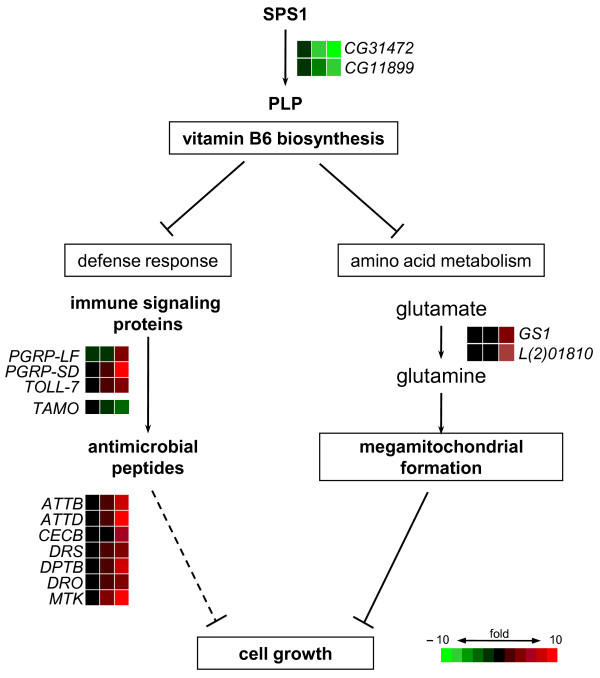
**A hypothetical model for molecular pathways regulated by SPS1**. A detailed explanation is provided in the Discussion. Molecular or cellular processes are marked with boxes. Proteins and molecules are in boldface letters. The expression levels of genes are marked with colors. The arrow and blocked line (┤) represent positive and negative regulation, respectively. The dashed line indicates that the effect was not proved experimentally.

A model for the molecular pathways regulated by SPS1 is summarized in Figure 5. SPS1 regulates the intracellular level of PLP by regulating the expression of genes responsible for PLP biosynthesis. Optimal levels of PLP do not induce defense response signaling and glutamine synthesis. However, low levels of PLP induce both defense signaling and glutamine synthesis. Once defense signaling is stimulated, genes responsible for the innate immune system, including AMPs, are activated. The activation of genes responsible for glutamine synthesis leads to megamitochondrial formation. The low level of intracellular PLP also leads to growth inhibition, presumably through induction of megamitochondrial formation and/or other biological processes. This hypothesis is supported by the observation of cell growth inhibition after the treatment of cells with 4-DPN (Figure 4A). However, it is not clear whether the growth inhibition is caused by the induction of both glutamine and AMP synthesis or one of these. In our previous study, it was found that conditions inducing megamitochondrial formation, such as the over expression of *GS1 *and *l(2)01810*, also resulted in cell growth inhibition [17]. But there is no report showing that the condition for the induction of defense system inhibits cell growth. Therefore, the inhibition of cell growth by AMP induction is represented as a dotted line in Figure 5.

Although SPS1 was found to regulate the biosynthesis of vitamin B6, the mechanism or signal pathway to which SPS1 is related has not been determined. Because SPS1 is localized to both plasma and nuclear membranes [38], it can be speculated that SPS1 regulates signal transduction by transducing signals on the plasma membrane or by transporting messengers or transcription factors through the nuclear membrane. The treatment of cell with 4-DPN or *SPS1 *knockdown induced the expression of *PGRP-SD *and *Toll-7*, which are involved in the Toll signaling pathway, and PGRP-LF, which is an activator of the IMD pathway (Figure 5). In addition, Tamo, which is a negative regulator for nuclear import of Dorsal, was found to be one of the down-regulated DEGs. These results strongly suggest that PLP, which is regulated by SPS1, participates in both the Toll and the IMD pathways.

Interestingly, *SPS1 *knockdown induced down-regulation of CG1753, which encodes cystathionine β-synthase (see Table 1). Cystathionine β-synthase catalyzes both L-cystathionine and L-selenocysteine synthesis [28]. Therefore, it seems that SPS1 regulates the synthesis of Sec indirectly by regulating the expression of Sec synthesizing enzymes.

## Conclusions

In this study, we predicted that vitamin B6 biosynthesis is the primary target of SPS1 by employing bioinformatics methods such as microarray and GO analyses and confirmed the prediction experimentally by showing that PLP levels were decreased by *SPS1 *knockdown and that the inhibition of PLP biosynthesis caused the same phenotypes as *SPS1 *knockdown.

## Methods

### Materials

Materials were purchased from the following sources: *Drosophila *Schneider cell line 2 (SL2) was purchased from Invitrogen, HyQ SFX-Insect medium from Hyclone, T3 Megascript kit from Ambion, RNeasy mini kit from Qiagen, GeneChip *Drosophila *genome 2.0 array from Affymetrix, SYBR Green mix from Applied Biosystems, TRIzol reagent from Invitrogen, Moloney murine leukemia virus reverse transcriptase from Super-Bio, 4-deoxypyridoxine hydrochloride from TCI, 5',6,6'-tetrachloro-1,1',3,3'-tetraethylbenzimidazolyl-carbocyanine iodide (JC-1) from Molecular Probes, and oligonucleotides from Cosmo Genetech. The sequences of oligos used for RT-PCR are listed in Additional File 6.

### SL2 cell culture and RNA interference

SL2 cell culture and preparation of double-stranded RNAs were carried out as described [17]. Briefly, for RNA interference, 0.25 × 10^6 ^cells were plated on a 24-well plate containing 0.5 ml of HyQ SFX-Insect medium. Four micrograms of dsRNAs were added directly to the medium and incubated for 48 hr and cells were split into appropriate culture dishes for further incubation and other experiments.

### Microarray experiment

Microarray experiments were performed using the GeneChip *Drosophila *genome 2.0 array. After the addition of double stranded RNAs targeting SPS1 to the culture medium, total RNA was extracted from SL2 cells treated with or without SPS1 dsRNA on day 1, 3 and 5 after treatment using the RNeasy mini kit according to the manufacturer's instructions. The cells that were not treated with any dsRNA were used as controls. The RNA quality was checked using Experion (Applied Biosystems) according to the manufacturer's instructions. Five micrograms of total RNAs were reverse transcribed with oligo-dT primer containing a T7 RNA polymerase promoter (TAATACGACTCACTATAGGG). Biotin-labeled cRNAs were generated from the cDNA sample by *in vitro *transcription with T7 RNA polymerase. The labeled cRNAs were fragmented to an average size of 35-200 bases by mild alkaline treatment at 94°C for 40 min. Fragmented cRNAs were hybridized with probes that are on GeneChip *Drosophila *genome 2.0 array, and the chips were washed and stained in the Affymetrix Fluidics Station 450 by following the procedures established by Affymetrix (Affymetrix GeneChip R Expression Analysis Technical Manual). The signals were scanned using the GeneChip Scanner 3000 7G (Affymetrix).

### Analysis of microarray data

The raw data were imported into Acuity 4.0 software (Molecular Devices, Inc.), and a background adjustment and normalization were performed using robust multichip average (RMA) and quantile methods, respectively, implemented in Acuity 4.0 software [39,40]. To identify differentially expressed genes (DEGs), a two-way analysis of variance (ANOVA) model was used and fitted using the R software http://www.r-project.org, as described by Park *et al *[41]. Two models were considered to identify DEGs. Model 1 contains group and time effects as well as their interactions. Model 1 allows the expression level of genes to change over time (days 1, 3 and 5) and these change patterns to differ between groups (control and knockdown). Model 2 includes only group and time effects assuming that the expression level of genes changes over time but these change patterns are the same between groups. From Model 1, DEGs were identified by the genes with significant interaction effects, while from Model 2 DEGs were identified by the genes with significant group effects. The p-values were adjusted by Westfall and Young's method [42]. The genes with adjusted p-values less than 0.1 were identified.

To classify DEGs according to their temporal expression pattern, DEGs were clustered using a self-organizing map (SOM) algorithm implemented in Acuity 4.0 [31]. The ratios of normalized log_2 _values of DEGs between *SPS1 *knockdown cells and control cells were used as input data and the SOM map size was set to 3 × 2. The ranges of expression ratios of DEGs within each cluster at each sampling date were displayed by box plot using R software. The interquartile ranges (IQRs) of each cluster were compared to select cluster(s) whose IQRs were significantly deviated. The criterion for determining clusters within which gene expressions were changed significantly was set to 0.75, i.e., when the interquartile range (IQR) of a cluster was larger than +0.75 or smaller than -0.75, the cluster was selected as significantly changed. This is because 0.75 is the threshold value to isolate clusters on day 3 (see Results for more details). The genes composing a cluster selected at the early stage (day 3) were defined as an early responding gene-set and those composing a cluster selected at the late stage (day 5) were defined as a late responding gene-set.

GO analysis was performed by BiNGO version 2.3 [33], which is plugged in Cytoscape [43]. Gene symbols of each gene-set were used as input data. The parameters were set as follows: assessment was set to overrepresentation, statistical test to binomial test, multiple testing correction to FWER correction, significance level to 0.05. Among GO evidence codes, inferred from electronically annotated (IEA) were discarded. The most significant pathway was predicted by considering the selected GO terms and visualized output.

### RT-PCR and quantitative real time RT-PCR

RT- PCR and real time PCR were carried out as described [17]. Briefly, total RNA was isolated from the cells using the TRIzol reagent. cDNAs were synthesized from total RNAs with Moloney murine leukemia virus reverse transcriptase and oligo (dT) primers according to the manufacturer's protocols. RT-PCR was performed with 0.1 μg of template total RNA and specific primers (Additional File 6). RT-PCR products were electrophoresed on a 2% agarose gel and visualized by ethidium bromide. For the measurement of relative mRNA levels of each gene, real time PCR was carried out using an ABI 7300 real time PCR system (Applied Biosystems) as follows. cDNAs were amplified using SYBR Green mix and specific primers for 40 cycles [initial incubation at 50°C for 2 min and then at 95°C for 10 min, and 40 cycles (95°C for 15 sec, 55°C for 1 min and 72°C for 1 min)]. Output data were obtained as *Ct *values using Sequence Detection Software (SDS) version 1.3 (7300 System, Applied Biosystems) and the differential mRNA expression of each gene between control and knockdown cell was calculated using the comparative *Ct *method [44]. RP49 mRNA, an internal control, was amplified along with the target genes, and the *Ct *value of RP49 used to normalize the expression of target genes.

### Measurement of intracellular PLP concentration

Cellular PLP levels were determined using the method previously described [45] with minor modifications. At day 5 after treatment with dsRNA or 4-DPN, cells were washed with phosphate buffered saline and harvested. Cells (6 × 10^7^) were lysed by resuspension in 600 μl of distilled water. Cell extracts were induced to produce the semicarbazon derivative of PLP as follows: 40 μl of 250 mg/ml of both semicarbazide and glycine were added into 500 μl of cell extracts or PLP standard. The mixture was vortexed and incubated at room temperature in the dark for 30 min. Proteins were then precipitated by adding 50 μl of 60% HClO_4 _into the mixture, and the solution was thoroughly mixed for 1 min. The solution was clarified by centrifugation for 10 min at 15,000 × g, and 30-50 μl of a 25% NaOH solution was added to the supernatant to achieve a pH between 3.0 and 5.0. HPLC was performed using a ZORBAX SB-C18 column (4.6 mm × 25 cm, PN 880975902) and an isocratic mobile phase consisting of 60 mM sodium phosphate (pH 6.5), 400 mg/l EDTA and 9.5% methanol at a flow-rate of 1 ml/min, and the derivatized PLP was quantified using a Waters™ 474 scanning fluorescence detector by setting excitation and emission wavelengths to 380 and 450 nm, respectively.

### Mitochondrial staining and confocal microscopy

Mitochondrial staining and confocal microscopy were carried out as described [17]. Briefly, SL2 cells (0.5 × 10^6^) were plated onto a chambered coverglass one day before staining. Cells were incubated with 1 μg/ml JC-1 for 30 min at 25°C, washed three times with HyQ-SFX-Insect medium and observed with a LSM510 confocal microscope (Carl Zeiss) at 512 × 512 pixel resolution through an X63 C-Apochromat objective. Excitation wavelengths for JC-1 aggregate and JC-1 monomer were 543 and 488 nm, respectively.

## Authors' contributions

MSS performed microarray experiments. KHL, EL and TP performed microarray data analysis. KHL performed temporal gene clustering and GO analysis. MSS, JYK and HKJ performed biochemical and cell biological experiments. BJL designed and directed the experiment and analysis. BAC, XMX, JMP and DLH advised and assisted in the interpretation of the results. KHL, MSS, DHL and BJL wrote the manuscript. All authors read and approved the manuscript.

## Supplementary Material

Additional file 1 **List of differentially expressed genes (DEGs)**. The list of DEGs whose expressions were changed more than 2 fold at least at one time point. Click here for file

Additional file 2 **Six Clusters of DEGs and gene-sets used for gene ontology analysis**. DEGs were grouped into six clusters and each cluster was classified as one of 3 gene-sets, after box-plotting the DEGs in each cluster. All the DEGs in each gene-set were used for gene ontology analysis. Click here for file

Additional file 3 **List of biological process terms selected by GO analysis with three gene-sets**. This table is an output obtained by running BinGO software showing genes and their GO biological process terms. The parameters used are described above the table. Click here for file

Additional file 4 **Hierarchical structures of GO terms obtained by performing early/down gene-set with different parameters**. Panels A and B were examples of hierarchical structures of GO terms obtained by analyzing early/down gene set with BinGO software. They showed similar results, although different parameters were used. Click here for file

Additional file 5 **Schematic diagram of vitamin B6 metabolic pathway**. The original vitamin B6 metabolic pathway diagram (collected from KEGG database) was modified by indicating DEGs and by showing their expression levels, after SPS1 was knocked down. Click here for file

Additional file 6 **Oligonucleotide sequences used as primers for RT-PCR or real-time PCR**. List of all oligonucleotides used for RT-PCR and real-time PCR. Click here for file
