# Supplementary material for: Drosophila selenophosphate synthetase 1 regulates vitamin B6 metabolism: prediction and confirmation
Source: BMC Genomics. 2011 Aug 24;12:426. doi: 10.1186/1471-2164-12-426 (PMC3218224; doi:10.1186/1471-2164-12-426)
Supplement: Additional file 1 — List of differentially expressed genes (DEGs). The list of DEGs whose expressions were changed more than 2 fold at least at one time point. [file 1471-2164-12-426-S1.PDF]

Additional File 1. List of differenctially expressed genes

	CG Symbol	Symbol	Name	ANOVA	Fold change			Gene Ontology	
				p-value	Day 1	Day 3	Day 5	Molecular Function	Biological Process
*	CG14196	CG14196		1.13E-03	0.98	1.06	2.15	secondary active monocarboxylate transmembrane transporter activity	transmembrane transport
*	CG7496	PGRP-SD	PGRP-SD	3.57E-03	0.96	1.73	11.73	peptidoglycan binding	defense response to Gram-positive bacterium
*	CG9042	Gpdh	Glycerol 3 phosphate dehydrogenase	3.87E-03	0.97	1.24	2.70	glycerol-3-phosphate dehydrogenase (NAD+) activity	triglyceride metabolic process
*	CG30059	CG30059		7.18E-03	0.96	1.14	1.43	N-acetylglucosamine-6-sulfatase activity	N-acetylglucosamine metabolic process
*	CG4926	Ror	Ror	1.21E-02	1.02	1.34	2.48	transmembrane receptor protein tyrosine kinase activity	central nervous system development
*	CG32185	CG32185		1.23E-02	1.06	2.33	12.82	_	_
*	CG1311	CG1311		2.64E-02	0.97	0.68	0.32	_	_
*	CG30046	CG30046		3.17E-02	0.95	1.18	2.04	_	_
*	CG14253	CG14253		6.36E-02	0.96	0.64	3.22	_	_
*	CG3413	wdp	windpipe	6.97E-02	0.97	1.59	3.04	_	_
*	CG6231	CG6231		7.63E-02	0.94	1.36	5.53	secondary active organic cation transmembrane transporter activity	transmembrane transport
$	CG31472	CG31472		2.85E-04	0.68	0.15	0.07	pyridoxamine-phosphate oxidase activity	pyridoxine biosynthetic process
$	CG32280	CG32280		1.58E-03	0.95	0.35	0.25	_	_
$	CG9505	CG9505		1.62E-03	1.08	1.82	6.59	metalloendopeptidase activity	proteolysis
$	CG12919	egr	eiger	3.40E-03	0.98	2.03	2.85	protein binding	immune response
$	CG5272	gnu	giant nuclei	3.87E-03	0.92	0.88	0.67	_	regulation of cell cycle
$	CG15526	CG15526		7.53E-03	1.01	4.38	9.68	_	_
$	CG1572	CG1572		8.40E-03	0.78	0.22	0.10	_	_
$	CG30456	CG30456		1.32E-02	1.06	2.19	3.75	Rho guanyl-nucleotide exchange factor activity	regulation of Rho protein signal transduction
$	CG31658	Nnf1b	Nnf1b	2.26E-02	0.93	0.45	0.18	_	mitotic metaphase plate congression
$	CG3625	CG3625		3.45E-02	0.93	0.42	0.11	_	_
$	CG14872	CG14872		4.20E-02	0.96	0.45	0.23	binding	_
$	CG6639	CG6639		5.55E-02	1.10	1.18	1.95	serine-type endopeptidase activity	proteolysis
	CG4398	CG4398		2.05E-04	0.97	0.65	0.14	_	_
	CG13795	CG13795		1.10E-03	0.88	0.77	0.33	neurotransmitter transporter activity	neurotransmitter transport
	CG1344	CG1344		1.36E-03	1.02	0.66	0.46	protein kinase activity	_
	CG3920	l(2)k16918	lethal (2) k16918	1.54E-03	0.88	0.70	0.33	_	_
	CG17610	grk	gurken	1.87E-03	0.78	0.45	0.27	epidermal growth factor receptor binding	maternal determination of dorsal/ventral axis
	CG10824	CG10824		2.44E-03	0.78	0.36	0.35	_	_
	CG32521	CG32521		2.63E-03	0.76	0.45	0.29	_	_
	CG2718	Gs1	Glutamine synthetase 1	3.54E-03	1.06	1.27	2.84	glutamate-ammonia ligase activity	glutamine metabolic process
	CG14079	CG14079		3.82E-03	1.00	0.56	0.43	_	_
	CG6204	CG6204		3.84E-03	0.97	0.68	0.43	_	_
	CG11822	nAcRbeta-21	nicotinic acetylcholine receptor beta 21C	3.95E-03	0.87	1.61	2.46	nicotinic acetylcholine-activated cation-selective channel activity	ion transport
	CG8588	pst	pastrel	4.33E-03	0.59	0.27	0.32	_	olfactory learning
	CG4250	CG4250		4.63E-03	1.09	1.55	6.66	_	_
	CG6137	aub	aubergine	5.02E-03	0.83	0.44	0.43	piRNA binding	regulation of metabolic process
	CG2086	drpr	draper	5.60E-03	1.10	1.31	2.31	protein binding	phagocytosis
	CG10810	Drs	Drosomycin	5.68E-03	0.97	1.35	2.33	_	defense response to fungus
	CG18104	arg	arginase	5.95E-03	1.05	1.64	3.42	arginase activity	arginine catabolic process to ornithine
	CG18528	CG18528		6.30E-03	0.92	0.56	0.35	GTPase activity	tRNA modification
	CG2200	CG2200		6.53E-03	0.85	0.55	0.39	dipeptidase activity	proteolysis

CG Symbol	Symbol	Name	ANOVA p-value	Fold change Day 1	Day 3	Day 5	Gene Ontology Molecular Function	Biological Process
CG17566	gammaTub37C	gamma-Tubulin at 37C	7.58E-03	0.84	0.30	0.19	GTP binding	microtubule-based process
CG1628	CG1628		7.68E-03	0.90	0.66	0.69	amino acid transmembrane transporter activity	mitochondrial ornithine transport
CG3779	numb	numb	7.79E-03	0.85	0.66	0.38	protein binding	regulation of developmental process
CG4057	tamo	tamo	8.73E-03	0.84	0.56	0.35	Ran GTPase binding	protein transport
CG18372	AttB	Attacin-B	8.94E-03	1.02	1.74	8.84	_	defense response to bacterium
CG6659	CG6659		9.78E-03	0.80	0.34	0.23	_	_
CG8175	Mtk	Metchnikowin	9.97E-03	1.08	3.38	25.02	_	defense response to fungus
CG33462	CG33462		1.09E-02	0.83	0.34	0.21	serine-type endopeptidase activity	proteolysis
CG1753	CG1753		1.15E-02	0.85	0.48	0.29	cystathionine beta-synthase activity	cysteine biosynthetic process from serine
CG12367	Hen 1		1.15E-02	0.95	0.71	0.43	O-methyltransferase activity	posttranscriptional gene silencing by RNA
CG7995	CG7995		1.19E-02	1.00	0.80	0.50	glycerol kinase activity	glycerol-3-phosphate metabolic process
CG8031	CG8031		1.19E-02	1.05	1.39	1.69	_	_
CG32625	CG32625		1.19E-02	1.19	1.41	5.08	_	_
CG5958	CG5958		1.23E-02	1.07	1.61	3.14	retinal binding	transport
CG3085	CG3085		1.26E-02	1.15	2.00	3.05	_	microtubule cytoskeleton organization
CG17259	CG17259		1.27E-02	0.95	0.84	0.80	serine-tRNA ligase activity	seryl-tRNA aminoacylation
CG17124	CG17124		1.27E-02	0.97	0.78	2.03	phosphoprotein phosphatase inhibitor activity	regulation of phosphorylation
CG33250	AlkB	AlkB	1.28E-02	0.90	0.52	0.40	oxidoreductase activity	_
CG8745	CG8745		1.28E-02	1.17	2.30	7.04	ornithine-oxo-acid transaminase activity	arginine catabolic process to glutamate
CG1218	CG1218		1.30E-02	0.87	0.65	0.40	_	_
CG8994	exu	exuperantia	1.33E-02	0.89	0.47	0.38	_	embryonic development via the syncytial blastoderm
CG9739	fz2	frizzled 2	1.38E-02	0.93	0.96	2.01	Wnt receptor activity	receptor-mediated endocytosis
CG8595	Toll-7	Toll-7	1.43E-02	0.92	1.31	2.03	transmembrane receptor activity	signal tranduction
CG6042	Cyp12a4	Cyp12a4	1.43E-02	1.04	0.80	2.09	electron carrier activity	response to insecticide
CG11899	CG11899		1.44E-02	0.90	0.68	0.36	O-phospho-L-serine:2-oxoglutarate aminotransferase activity	pyridoxine biosynthetic process
CG4947	Tgt	tRNA-guanine transglycosylase	1.44E-02	0.97	0.70	0.44	queuine tRNA-ribosyltransferase activity	queuosine biosynthetic process
CG9008	CG9008		1.44E-02	0.74	0.47	0.51	isomerase activity	carbohydrate metabolic process
CG32446	Atox1		1.51E-02	0.86	0.43	0.21	metal ion binding	metal ion transporter
CG15083	CG15083		1.52E-02	0.93	0.81	0.44	_	_
CG17559	dnt	doughnut on 2	1.55E-02	0.90	0.74	0.67	transmembrane receptor protein tyrosine kinase activity	signal transduction
CG4500	CG4500		1.55E-02	1.04	0.86	2.79	long-chain fatty acid-CoA ligase activity	mesoderm development
CG10248	Cyp6a8	Cytochrome P450-6a8	1.55E-02	0.88	0.76	2.94	alkane 1-monooxygenase activity	insecticide metabolic process
CG1878	CecB	Cecropin B	1.55E-02	0.98	1.15	4.79	_	defense response to Gram-negative bacterium
CG9460	Spn42De	Serpin 42De	1.58E-02	0.85	0.68	0.60	serine-type endopeptidase inhibitor activity	_
CG9331	CG9331		1.58E-02	0.99	0.72	2.24	NAD or NADH binding	metabolic process
CG14346	CG14346		1.68E-02	0.89	0.77	0.48	_	_
CG4786	Rcd2	Reduction in Cnn dots 2	1.68E-02	0.90	0.50	0.89	_	centriole replication
CG4210	CG4210		1.74E-02	0.84	0.32	0.15	N-acetyltransferase activity	metabolic process
CG17186	CG17186		1.88E-02	1.07	1.54	1.73	zinc ion binding	_
CG16888	CG16888		1.88E-02	1.07	1.77	3.15	_	_
CG4502	CG4502		1.89E-02	0.85	0.50	0.40	acid-amino acid ligase activity	post-translational protein modification
CG40293	Stlk	Ste20-like kinase	1.93E-02	0.91	0.77	0.62	protein serine/threonine kinase activity	protein amino acid phosphorylation

CG Symbol	Symbol	Name	ANOVA	Fold change			Gene Ontology	
			p-value	Day 1	Day 3	Day 5	Molecular Function	Biological Process
CG6124	eater	eater	1.93E-02	0.96	1.17	10.24	bacterial cell surface binding	phagocytosis
CG12316	CG12316		1.96E-02	1.21	1.19	1.57	_	_
CG9681	PGRP-SB1	PGRP-SB1	1.99E-02	0.93	0.38	0.94	peptidoglycan binding	immune response
CG34008	CG34008		2.00E-02	1.00	0.49	0.13	_	_
CG4259	CG4259		2.00E-02	0.99	0.65	7.87	serine-type endopeptidase activity	proteolysis
CG3570	CG3570		2.01E-02	0.90	0.71	0.43	_	_
CG12608	CG12608		2.08E-02	0.92	0.63	0.44	_	_
CG2715	Syx4	Syntaxin 4	2.21E-02	0.92	0.41	0.30	SNAP receptor activity	inter-male aggressive behavior
CG15399	CG15399		2.23E-02	0.91	0.42	0.23	_	_
CG3770	CG3770		2.26E-02	0.87	0.78	0.41	_	establishment and or maintenance of cell polarity
CG3792	CG3792		2.26E-02	1.06	1.28	1.40	_	_
CG13315	CG13315		2.29E-02	0.95	0.37	4.95	_	_
CG5144	CG5144		2.32E-02	1.03	0.36	0.67	arginine kinase activity	_
CG10063	CG10063		2.61E-02	0.90	0.30	0.25	_	_
CG6383	crb	crumbs	2.65E-02	0.99	0.68	0.43	protein kinase C binding	system development; biological regulation; cell junction organization
CG32706	CG32706		2.68E-02	0.87	0.49	0.43	nucleotide binding	_
CG17129	CG17129		2.73E-02	0.93	0.57	0.39	_	_
CR8687	Cyp6a14	Cyp6a14	2.73E-02	0.97	1.04	2.03	electron carrier activity	oxidation reduction
CG17721	CG17721		2.74E-02	0.91	0.63	0.41	zinc ion binding	_
CG31431	CG31431		2.79E-02	0.89	0.49	0.59	fibroblast growth factor receptor activity	fibroblast growth factor receptor signaling pathway
CG16790	CG16790		2.91E-02	0.97	0.75	0.47	protein binding	RNA metabolic process
CG6863	tok	tolkin	2.91E-02	0.91	1.72	3.19	metalloendopeptidase activity	motor axon guidance
CG32170	CG32170		2.96E-02	0.97	1.09	3.55	transition metal ion binding	oxidation reduction
CG12283	kek1	kekkon-1	3.01E-02	0.81	0.56	0.45	epidermal growth factor binding	negative regulation of epidermal growth factor receptor signaling pathway
CG32043	CG32043		3.04E-02	0.81	0.71	0.44	_	_
CG6199	CG6199		3.04E-02	1.01	1.32	2.05	procollagen-lysine 5-dioxygenase activity	oxidation reduction
CG9733	CG9733		3.13E-02	0.94	0.38	1.77	serine-type endopeptidase activity	proteolysis
CG6965	mthl5	methuselah-like 5	3.22E-02	0.89	0.44	0.25	G-protein coupled receptor activity	G-protein coupled receptor protein signaling pathway
CG12390	dare	defective in the avoidance of repellents	3.33E-02	1.05	0.53	0.21	NADPH-adrenodoxin reductase activity	steroid biosynthetic process
CG9623	if	inflated	3.33E-02	1.10	1.19	2.14	receptor activity	biological regulation
CG3960	CG3960		3.39E-02	0.77	0.59	0.33	actin binding	mesoderm development
CG3950	CG3950		3.39E-02	0.88	0.68	0.39	actin binding	mesoderm development
CG3074	CG3074		3.39E-02	0.99	0.89	2.05	cysteine-type endopeptidase activity	proteolysis
CG3884	CG3884		3.39E-02	0.97	1.19	2.38	_	_
CG32985	CG32985		3.42E-02	0.85	0.26	0.07	catalytic activity	metabolic process
CG18410	Ude	Uracil-DNA degrading factor	3.50E-02	0.89	0.58	0.49	DNA binding	pupation
CG5210	CG5210		3.56E-02	1.10	1.82	2.56	chitin binding	cuticle chitin catabolic process (Chit)
CG4437	PGRP-LF	Peptidoglycan recognition protein LF	3.56E-02	0.76	0.75	3.82	peptidoglycan binding	innate immune response
CG7629	AttD	Attacin-D	3.66E-02	1.00	1.60	10.27	_	antibacterial humoral response
CG12002	Pxn	Peroxidasin	3.69E-02	0.98	1.74	5.17	peroxidase activity	response to oxidative stress
CG8936	Arpc3B	Arpc3B	3.73E-02	0.75	0.41	0.42	actin binding	actin filament organization
CG42280	ome	omega	3.76E-02	0.93	1.42	5.45	dipeptidyl-peptidase activity	proteolysis
CG5304	l(2)01810	lethal (2) 01810	3.79E-02	1.12	1.98	3.91	high affinity inorganic phosphate:sodium symporter activity	transmembrane transporter
CG12880	CG12880		3.80E-02	0.69	0.72	0.25	_	_
CG2100	CG2100		3.91E-02	0.90	0.67	0.43	polynucleotide adenylyltransferase activity	RNA processing

CG Symbol	Symbol	Name	ANOVA	Fold change			Gene Ontology	
			p-value	Day 1	Day 3	Day 5	Molecular Function	Biological Process
CG14229	CG14229		3.99E-02	0.94	0.66	0.49	_	_
CG2177	CG2177		4.04E-02	0.88	0.55	0.39	metal ion transmembrane transporter activity	transmembrane transport
CG31274	CG31274		4.07E-02	0.94	0.52	0.51	_	_
CG31075	CG31075		4.07E-02	1.03	1.27	2.33	aldehyde dehydrogenase (NAD) activity	pyruvate metabolic process
CG4472	Idgf1	Imaginal disc growth factor 1	4.12E-02	0.89	1.23	2.90	imaginal disc growth factor activity	imaginal disc development
CG2003	CG2003		4.13E-02	0.95	1.25	2.32	_	_
CG32680	spri	sprint	4.13E-02	0.94	1.25	2.70	RasGTPase binding	border folicle cell migration
CG6018	CG6018		4.13E-02	1.06	1.82	3.29	carboxylesterase activity	_
CG34033	CG34033		4.15E-02	0.88	0.73	0.43	_	_
CG2669	CG2669	humpty dumpty	4.23E-02	1.01	0.66	0.38	_	cell proliferation
CG2794	CG2794		4.23E-02	1.03	1.43	1.59	phosphotransferase activity	_
CG10160	ImpL3	Ecdysone-inducible gene L3	4.33E-02	1.00	0.65	0.36	L-lactate dehydrogenase activity	glycolysis
CG33134	debcl	death executioner Bcl-2 homologue	4.49E-02	0.94	0.65	0.44	_	negative regulation of neuron apoptosis
CG10424	CG10424		4.55E-02	0.95	0.64	0.37	_	_
CG8782	Oat	Ornithine aminotransferase precursor	4.55E-02	0.89	0.91	3.12	ornithine-oxo-acid transaminase activity	ornithine metabolic process
CG6043	CG6043		4.59E-02	1.04	1.00	2.06	_	_
CG10794	DptB	Diptericin B	4.69E-02	1.02	1.98	8.90	_	antibacterial humoral response
CG3961	CG3961		4.71E-02	0.92	0.87	0.42	long-chain-fatty-acid-CoA ligase activity	metabolic process
CG5630	CG5630		4.73E-02	1.09	1.43	2.52	_	_
CG6612	Adk3	Adenylate kinase-3	4.73E-02	1.11	1.15	2.60	nucleoside triphosphate adenylate kinase activity	ADP biosynthetic process
CG12883	CG12883		4.82E-02	0.98	0.56	0.25	_	_
CG33503	Cyp12d1-d	Cyp12d1-d	4.89E-02	0.98	1.25	2.21	electron carrier activity	oxidation reduction
CG7381	CG7381		4.91E-02	0.99	0.67	0.48	_	_
CG7903	CG7903		4.91E-02	1.04	1.27	1.45	mRNA binding	_
CG10564	Ac78C	Adenylyl cyclase 78C	4.93E-02	0.95	1.23	3.77	adenylate cyclase activity	response to sucrose stimulus
CG4711	squ	squash	4.94E-02	0.96	0.65	0.45	_	gene silencing by RNA
CG30489	Cyp12d1-p	Cyp12d1-p	5.01E-02	0.99	1.18	2.23	electron carrier activity	oxidation reduction
CG17725	Pepck	Phosphoenolpyruvate carboxykinase	5.08E-02	1.11	1.23	4.88	phosphoenolpyruvate carboxykinase (GTP) activity	gluconeogenesis
CG4559	Idgf3	Imaginal disc growth factor 3	5.11E-02	1.01	1.27	2.04	imaginal disc growth factor activity	imaginal disc development
CG33468	CG33468		5.14E-02	1.05	0.66	2.30	_	_
CG6126	CG6126		5.18E-02	0.98	1.40	2.32	organic cation transmembrane transporter activity	transmembrane transport
CG14680	Cyp12e1	Cyp12e1	5.34E-02	1.06	1.47	2.18	electron carrier activity	oxidation reduction
CG30484	CG30484		5.36E-02	0.70	0.82	3.43	_	_
CG17834	CG17834		5.39E-02	0.90	0.39	0.98	_	_
CG2984	Pp2C1	Protein phosphatase 2C	5.39E-02	1.15	1.06	1.38	protein serine/threonine phosphatase activity	protein amino acid dephosphorylation
CG3424	path	pathetic	5.39E-02	0.95	1.21	2.35	acid transmembrane transporter activity	growth
CG13117	CG13117		5.40E-02	0.78	0.44	0.43	_	_
CG32714	CG32714		5.55E-02	1.09	1.16	2.11	_	_
CG7123	LanB1	LanB1	5.58E-02	1.02	1.16	2.08	_	organ development; cell migration
CG8051	CG8051		5.61E-02	1.08	1.53	2.18	secondary active monocarboxylate transmembrane transporter activity	transmembrane transport
CG31142	CG31142		5.62E-02	1.02	0.74	0.27	_	_
CG9102	bab2	bab2	5.62E-02	0.92	1.21	2.24	protein binding	imaginal disc-derived leg morphogenesis
CG8846	Thor	Thor	5.68E-02	1.09	1.01	2.41	eukaryotic initiation factor 4E binding	immune response
CG12643	CG12643		5.69E-02	1.01	0.94	0.38	_	_

CG Symbol	Symbol	Name	ANOVA	Fold change			Gene Ontology	
			p-value	Day 1	Day 3	Day 5	Molecular Function	Biological Process
CG40498	CG40498		5.72E-02	1.08	1.41	2.31	_	_
CG7504	CG7504		5.76E-02	0.93	0.72	0.49	ATP-dependent RNA helicase activity	_
CG7523	CG7523		5.76E-02	1.02	1.42	1.82	_	_
CG6127	Ser	Serrate	6.00E-02	1.16	1.42	2.40	protein binding	biological regulation
CG4953	CG4953		6.01E-02	0.94	0.74	0.52	_	_
CG9968	Anxb11	Annexin B11	6.05E-02	1.03	1.45	2.09	actin binding	regulation of cell shape
CG4615	CG4615		6.13E-02	0.90	0.78	0.79	_	phagocytosis
CG4330	CG4330		6.19E-02	0.93	0.62	0.42	high affinity inorganic phosphate:sodium symporter activity	transmembrane transport
CG17181	CG17181		6.32E-02	0.94	0.50	0.29	zinc ion binding	_
CG2913	yin	yin	6.44E-02	0.92	1.61	2.66	proton-dependent oligopeptide secondary active transmembrane transporter activity	oligopeptide transport
CG9338	CG9338		6.45E-02	0.91	0.49	0.77	_	_
CG2259	Gclc	Glutamate-cysteine ligase catalytic	6.49E-02	0.94	0.72	0.92	glutamate-cysteine ligase activity	glutathione biosynthetic process
CG11299	Sesn	Sestrin	6.49E-02	1.13	1.78	2.99	_	negative regulation of cell growth (mitochondrion
CG10816	Dro	Drosocin	6.49E-02	1.06	1.61	3.81	_	defense response to Gram-positive bacterium
CG4475	Idgf2	Imaginal disc growth factor 2	6.58E-02	1.00	1.24	2.54	imaginal disc growth factor activity	imaginal disc development
CG5165	Pgm	Phosphogluconate mutase	6.70E-02	1.00	0.41	0.17	phosphoglycerate mutase activity	glycogen biosynthetic process
CG17905	ChLD3	ChLD3	6.70E-02	0.92	0.55	0.38	hydrolase activity	chitin metabolic process
CG12840	Tsp42El	Tetraspanin 42El	6.81E-02	0.82	0.32	0.37	_	_
CG12018	CG12018		6.84E-02	0.96	0.71	0.50	DNA-directed DNA polymerase activity	cellular response to DNA damage stimulus
CG13654	CG13654		6.94E-02	1.00	1.11	2.37	_	_
CG18412	ph-p	polyhomeotic proximal	6.96E-02	0.86	0.73	0.41	chaperone binding	central nervous system neuron development
CG2065	CG2065		6.96E-02	0.87	0.37	0.81	affinity inorganic phosphate:sodium symporter activity	transmembrane transport
CG11513	armi	armitage	6.98E-02	0.98	0.71	0.42	DNA helicase activity	nuclear-transcribed mRNA catabolic process
CG10999	CG10999		6.98E-02	0.92	0.66	0.60	_	_
CG4338	CG4338		7.02E-02	0.97	0.75	0.55	_	_
CG33521	CG33521		7.02E-02	0.99	1.43	2.14	zinc ion binding	_
CG5126	CG5126		7.21E-02	0.96	0.75	0.43	_	_
CG3132	Ect3	Beta-galactosidase	7.21E-02	0.97	1.25	5.25	beta-galactosidase activity	autophagic cell death
CG15922	CG15922		7.22E-02	0.95	0.74	0.51	_	_
CG8157	CG8157		7.29E-02	0.83	0.59	0.54	_	_
CG8147	CG8147		7.29E-02	0.95	1.35	2.80	alkaline phosphatase activity	metabolic process
CG1318	Hexo1	Hexosaminidase 1	7.44E-02	0.92	0.53	0.24	beta-N-acetylglucosaminidase activity	carbohydrate metabolic process
CG12014	CG12014		7.46E-02	0.77	0.23	0.10	iduronate-2-sulfatase activity	metabolic process
CG15678	pirk	poor Imd response upon knock-in	7.56E-02	0.95	0.58	3.71	receptor binding	negative regulation of innate immune response
CG1598	CG1598		7.61E-02	0.95	0.93	0.83	arsenite-transporting ATPase activity	cellular metal ion homeostasis
CG15893	CG15893		7.77E-02	0.96	0.31	0.54	_	_
CG3348	CG3348		7.79E-02	0.96	0.70	2.97	chitin binding	chitin metabolic process
CG9641	CG9641		7.85E-02	0.90	0.61	0.34	_	_
CG5008	GNBP3	Gram-negative bacteria binding protein 3	7.93E-02	0.88	0.42	0.29	pattern recognition receptor activity	response to fungus
CG6854	CTPsyn	CTP synthase	7.95E-02	0.89	0.74	0.32	CTP synthase activity	CTP biosynthetic process
CG4531	argos	argos	8.05E-02	0.59	0.69	0.19	receptor antagonist activity	wing disc morphogenesis
CG1787	Hexo2	Hexosaminidase 2	8.37E-02	0.94	0.65	0.29	beta-N-acetylglucosaminidase activity	negative regulation of growth of symbiont in host

CG Symbol	Symbol	Name	ANOVA	Fold change			Gene Ontology	
			p-value	Day 1	Day 3	Day 5	Molecular Function	Biological Process
CG1962	CG1962		8.39E-02	0.97	0.71	0.46	_	_
CG5123	W	Wrinkled	8.44E-02	0.88	1.45	2.25	_	biological regulation
CG9453	Spn4	Serine protease inhibitor 4	8.56E-02	1.08	1.09	2.03	serine-type endopeptidase inhibitor activity	negative regulation of peptide hormone processing
CG13602	CG13602		8.91E-02	1.02	0.40	0.44	_	_
CG14629	CG14629		9.20E-02	1.01	1.82	6.08	_	_
CG9471	CG9471		9.22E-02	0.97	0.76	0.46	NADPH dehydrogenase activity	metabolic process
CG10249	CG10249		9.36E-02	1.21	1.06	2.27	_	_
CG15078	Mctp	Multiple C2 domain and transmembrane region protein	9.38E-02	0.92	0.45	0.13	_	_
CG9154	CG9154		9.39E-02	1.03	1.66	1.46	methyltransferase activity	methylation
CG4501	bgm	bubblegum	9.39E-02	0.95	0.90	2.58	long-chain-fatty-acid-CoA ligase activity	long-chain fatty acid metabolic process
CG5535	CG5535		9.44E-02	0.93	0.54	0.16	amino acid transmembrane transporter activity	amino acid transport
CG6871	Cat	Catalase	9.54E-02	1.10	1.45	2.55	catalase activity	response to hydrogen peroxide
CG13907	CG13907		9.56E-02	1.07	2.15	2.92	secondary active monocarboxylate transmembrane transporter activity	transmembrane transport
CG4026	IP3K1	Inositol 1,4,5-triphosphate kinase 1	9.64E-02	1.05	0.93	2.25	inositol trisphosphate 3-kinase activity	response to oxidative stress
CG31151	wge	winged eye	9.66E-02	0.93	0.66	0.60	DNA binding	_
CG18550	yellow-f	yellow-f	9.66E-02	1.01	1.02	2.85	dopachrome isomerase activity	indole derivative biosynthetic process
CG31975	CG31975		9.67E-02	0.94	0.59	0.28	_	_
CG7737	CG7737		9.74E-02	0.88	0.71	0.43	_	_
CG5793	CG5793		9.78E-02	1.01	1.26	1.26	catalytic activity	metabolic process
CG6006	CG6006		9.78E-02	0.82	1.15	3.34	transporter activity	transmembrane transport
CG5322	CG5322		9.80E-02	1.11	1.41	6.18	alpha-mannosidase activity	mannose metabolic process
CG2604	CG2604		9.83E-02	0.95	0.78	0.49	catalytic activity	metabolic process
CG13077	CG13077		9.84E-02	0.90	1.71	6.10	_	_

Genes whose expression levels changed by group, time and their interaction effects (ANOVA model 1) are marked with an asterisk (*), and genes selected by both model 1 and 2 are with $. Other genes are selected by only group and time effects (ANOVA model 2)
