# Supplementary material for: Drosophila selenophosphate synthetase 1 regulates vitamin B6 metabolism: prediction and confirmation
Source: BMC Genomics. 2011 Aug 24;12:426. doi: 10.1186/1471-2164-12-426 (PMC3218224; doi:10.1186/1471-2164-12-426)
Supplement: Additional file 2 — Six Clusters of DEGs and gene-sets used for gene ontology analysis. DEGs were grouped into six clusters and each cluster was classified as one of 3 gene-sets, after box-plotting the DEGs in each cluster. All the DEGs in each gene-set were used for gene ontology analysis. [file 1471-2164-12-426-S2.PDF]

Additional File 2. Clusters of DEGs and gene-sets used for gene ontology analysis

				Early / down gene-set				
	Late / up gene-set				Late / down gene-set			
Cluster No.	1	2		3	4	5	6	
Gene No.	33 genes	77 genes		9 genes	12 genes	27 genes	80 genes	
Gene Symbol	AttB	Ac78C	CG5210	CG1962	argos	Atox1	AlkB	CG3950
	AttD	Adk3	CG7903	CG2065	aub	CG10424	armi	CG3960
	CecB	Anxb11	CG8031	CG31274	CG10824	CG11899	Arpc3B	CG3961
	CG13077	arg	CG8051	CG31975	CG13602	CG12014	CG10063	CG4330
	CG15526	bab2	CG8147	CG33468	CG15893	CG1218	CG10999	CG4338
	CG30484	bgm	CG9154	CG9733	CG17834	CG12643	CG12018	CG4502
	CG3085	Cat	CG9331	Gclc	CG31431	CG1572	CG12608	CG4615
	CG32170	CG10249	Cyp12a4	PGRP-SB1	CG5144	CG1753	CG12880	CG4953
	CG32185	CG12316	Cyp12d1-d	Pp2C1	CG9008	CG31472	CG12883	CG5126
	CG32625	CG13315	Cyp12d1-p		CG9338	CG32280	CG1311	CG6204
	CG32714	CG13654	Cyp12e1		pst	CG32985	CG13117	CG6659
	CG3348	CG13907	Cyp6a14		Tsp42El	CG33462	CG1344	CG7381
	CG4250	CG14196	Dro			CG34008	CG13795	CG7504
	CG4259	CG14253	drpr			CG3625	CG14079	CG7737
	CG5322	CG14629	Drs			CG4210	CG14229	CG7995
	CG6006	CG16888	egr			CG4398	CG14346	CG8157
	CG6018	CG17124	fz2			CG5535	CG14872	CG9471
	CG6231	CG17186	Gpdh			dare	CG15083	CG9641
	CG8745	CG2003	Gs1			gammaTub37C	CG15399	ChLD3
	CG9505	CG2794	Idgf1			Hen 1	CG15922	crb
	Cyp6a8	CG30046	Idgf2			Hexo1	CG1598	CTPsyn
	DptB	CG30059	Idgf3			l(2)k16918	CG1628	debcl
	eater	CG30456	if			Mctp	CG16790	dnt
	Ect3	CG3074	IP3K1			mthl5	CG17129	exu
	Mtk	CG31075	l(2)01810			Nnf1b	CG17181	GNBP3
	Oat	CG33521	LanB1			numb	CG17259	gnu
	ome	CG3792	nAcRbeta-21C			Pgm	CG17721	grk
	Pepck	CG3884	path				CG18528	Hexo2
	PGRP-LF	CG40498	Ror				CG2100	ImpL3
	PGRP-SD	CG4500	Ser				CG2177	kek1
	pirk	CG5630	Spn4				CG2200	ph-p
	Pxn	CG5793	Thor				CG2604	Rcd2
	wdp	CG5958	tok				CG2669	Spn42De
		CG6043	Toll-7				CG31142	squ
		CG6126	W				CG32043	Stlk
		CG6199	yellow-f				CG32521	Syx4
		CG6639	yin				CG32706	tamo
		CG7523					CG34033	Tgt
		Sesn					CG3570	Ude
		Spri					CG3770	wge
