# Supplementary material for: Drosophila selenophosphate synthetase 1 regulates vitamin B6 metabolism: prediction and confirmation
Source: BMC Genomics. 2011 Aug 24;12:426. doi: 10.1186/1471-2164-12-426 (PMC3218224; doi:10.1186/1471-2164-12-426)
Supplement: Additional file 3 — List of biological process terms selected by GO analysis with three gene-sets. This table is an output obtained by running BinGO software showing genes and their GO biological process terms. The parameters used are described above the table. [file 1471-2164-12-426-S3.PDF]

Additional File 3. List of biological process terms selected by GO analysis with three gene-sets

Discarded evidence codes : IEA
Overrepresentation
Selected statistical test : Binomial test
Selected correction : Bonferroni Family-Wise Error Rate (FWER) correction
Selected significance level : 0.05
Testing option : Use whole annotation as reference set

Gene-set	GO ID	p-value	corr. p-value	selected gene No.	genes No. in GO term	Annotated gene No. in gene-set	reference gene No.	GO term (Biological process)	Genes in test set
early/down	42816	5.29E-05	2.48E-02	2	3	26	7410	vitamin B6 biosynthetic process	*CG11899, CG31472*
	42819	5.29E-05	2.48E-02	2	3	26	7410	vitamin B6 metabolic process	*CG11899, CG31472*
	8615	5.29E-05	2.48E-02	2	3	26	7410	pyridoxine biosynthetic process	*CG11899, CG31472*
	8614	5.29E-05	2.48E-02	2	3	26	7410	pyridoxine metabolic process	*CG11899, CG31472*
late/up	9617	8.83E-11	6.22E-08	10	71	53	7410	response to bacterium	*CecB, PGRP-SD, Mtk, egr, pirk, Dro, AttB, DptB, AttD, Drs*
	42742	7.41E-10	5.22E-07	9	63	53	7410	defense response to bacterium	*CecB, PGRP-SD, Mtk, egr, Dro, AttB, DptB, AttD, Drs*
	6952	9.85E-10	6.95E-07	12	157	53	7410	defense response	*CecB, PGRP-LF, W, PGRP-SD, Mtk, egr, Dro, AttB, DptB, AttD, Drs, Toll-7*
	19731	1.14E-09	8.06E-07	7	27	53	7410	antibacterial humoral response	*CecB, Mtk, Dro, AttB, DptB, AttD, Drs*
	6955	6.69E-09	4.72E-06	11	147	53	7410	immune response	*CecB, PGRP-LF, W, PGRP-SD, Mtk, egr, Dro, AttB, DptB, AttD, Drs*
	9308	8.80E-09	6.21E-06	9	151	53	7410	amine metabolic process	*yellow-f, Gs1, PGRP-LF, W, PGRP-SD, arg, Oat, CG8745, Drs*
	2376	1.09E-08	7.68E-06	11	195	53	7410	immune system process	*CecB, PGRP-LF, W, PGRP-SD, Mtk, egr, Dro, AttB, DptB, AttD, Drs*
	51707	2.73E-08	1.93E-05	10	130	53	7410	response to other organism	*CecB, PGRP-SD, Mtk, egr, pirk, Dro, AttB, DptB, AttD, Drs*
	9607	4.15E-08	2.93E-05	10	136	53	7410	response to biotic stimulus	*CecB, PGRP-SD, Mtk, egr, pirk, Dro, AttB, DptB, AttD, Drs*
	19752	1.16E-07	8.17E-05	8	152	53	7410	carboxylic acid metabolic process	*yellow-f, Gs1, Cyp6a8, arg, Pepck, Oat, CG8745, Drs*
	43436	1.16E-07	8.17E-05	8	152	53	7410	oxoacid metabolic process	*yellow-f, Gs1, Cyp6a8, arg, Pepck, Oat, CG8745, Drs*
	6082	1.16E-07	8.17E-05	8	152	53	7410	organic acid metabolic process	*yellow-f, Gs1, Cyp6a8, arg, Pepck, Oat, CG8745, Drs*
	42180	2.74E-07	1.93E-04	8	167	53	7410	cellular ketone metabolic process	*yellow-f, Gs1, Cyp6a8, arg, Pepck, Oat, CG8745, Drs*
	51704	3.52E-07	2.48E-04	11	269	53	7410	multi-organism process	*CecB, W, PGRP-SD, Mtk, egr, pirk, Dro, AttB, DptB, AttD, Drs*
	19730	9.40E-07	6.63E-04	7	73	53	7410	antimicrobial humoral response	*CecB, Mtk, Dro, AttB, DptB, AttD, Drs*
	44106	9.46E-07	8.67E-04	7	107	53	7410	cellular amine metabolic process	*yellow-f, Gs1, W, arg, Oat, CG8745, Drs*
	50829	2.67E-06	1.88E-03	5	30	53	7410	defense response to Gram-negative bacterium	*CedB, Mtk, egr, Dro, Drs*
	6959	3.20E-06	2.26E-03	7	88	53	7410	humoral immune response	*CecB, Mtk, Dro, AttB, DptB, AttD, Drs*
	6519	3.54E-06	3.50E-03	6	128	53	7410	cellular amino acid and derivative metabolic process	*yellow-f, Gs1, W, arg, Oat, CG8745*
	6950	5.60E-06	7.95E-03	12	485	53	7410	response to stress	*PGRP-LF, CecB, egr, AttB, AttD, W, PGRP-SD, Mtk, Dro, DptB, Toll-7, Drs*
	6520	3.54E-05	2.49E-02	6	86	53	7410	cellular amino acid metabolic process	*yellow-f, Gs1, arg, Oat, CG8745, Drs*
	50830	3.89E-05	2.74E-02	4	26	53	7410	defense response to Gram-positive bacterium	*CecB, PGRP-SD, Mtk, Dro*
	50832	5.17E-05	3.65E-02	4	28	53	7410	defense response to fungus	*CecB, Mtk, Dro, Drs*
	50896	1.72E-05	3.91E-02	15	1138	53	7410	response to stimulus	*CecB, PGRP-LF, egr, pirk, AttB, AttD, W, PGRP-SD, Cyp6a8, Mtk, Dro, DptB, Cyp12a4, Toll-7, Drs*
	9620	6.74E-05	4.75E-02	4	30	53	7410	response to fungus	*CecB, Mtk, Dro, Drs*
late/down	No significant biological processes are selected.								
