# Supplementary material for: Drosophila selenophosphate synthetase 1 regulates vitamin B6 metabolism: prediction and confirmation
Source: BMC Genomics. 2011 Aug 24;12:426. doi: 10.1186/1471-2164-12-426 (PMC3218224; doi:10.1186/1471-2164-12-426)
Supplement: Additional file 4 — Hierarchical structures of GO terms obtained by performing early/down gene-set with different parameters. Panels A and B were examples of hierarchical structures of GO terms obtained by analyzing early/down gene set with BinGO software. They showed similar results, although different parameters were used. [file 1471-2164-12-426-S4.PDF]

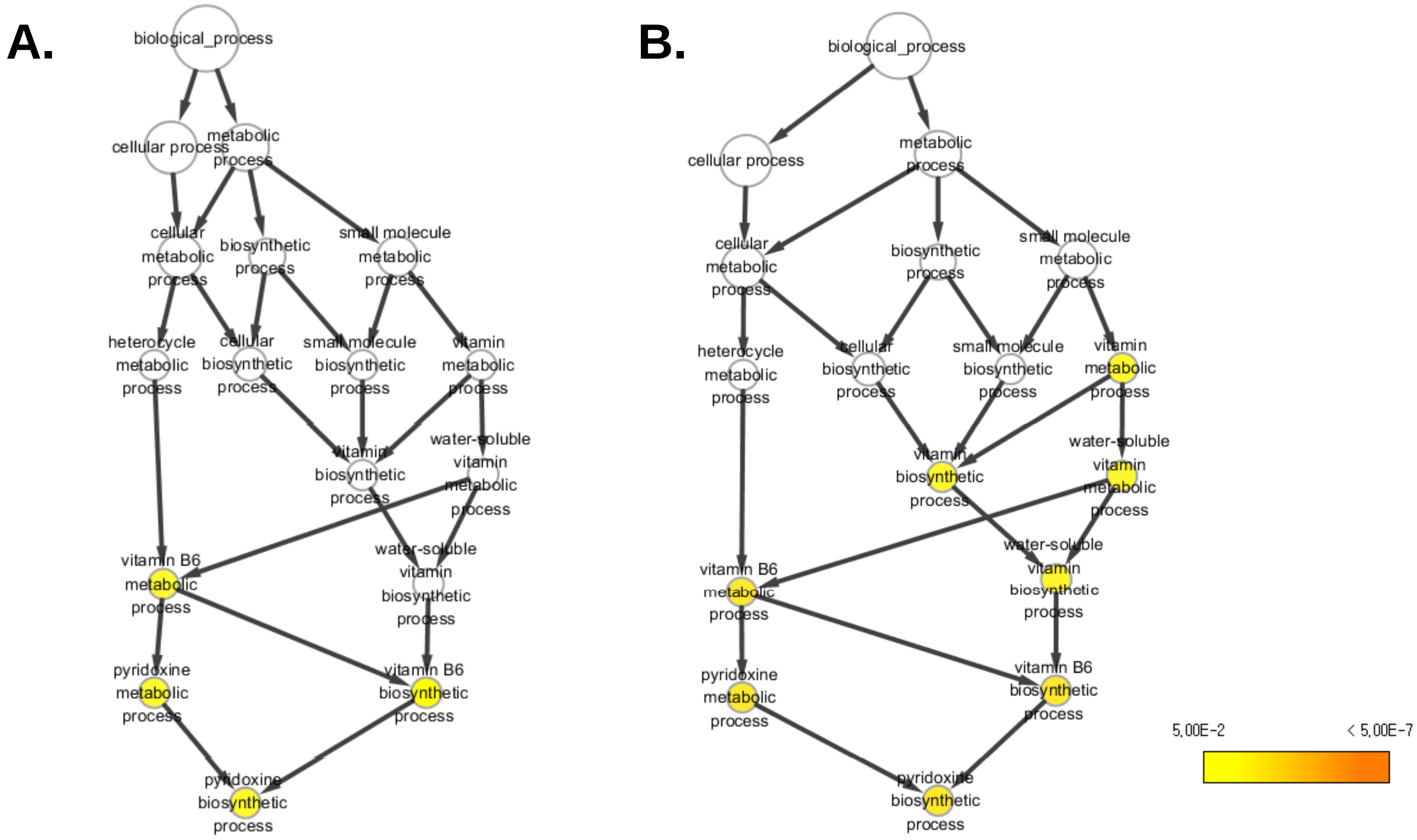


**Additional File 4. Hierarchical structures of GO terms obtained by performing GO analysis with early/down gene-set in two different parameters**

Early/down gene-set was analyzed for significant GO terms using BinGO software as described in Materials and Methods, and the outputs were redrawn using by Cytoscape. All parameters were the same except the statistical model and multiple testing corrections; **A.** Binomial test and FWER (Bonferroni Family-Wise Error Rate) correction were used. **B.** Hypergeometric test and FDR (Benjamini and Hochberg False Discovery) correction was used. The circles and arrows represent nodes for GO terms and parent-child relations, respectively. The p-value of each node is expressed by the intensity of color. As the color becomes darker, the p-value decreases.
