# Supplementary material for: Drosophila selenophosphate synthetase 1 regulates vitamin B6 metabolism: prediction and confirmation
Source: BMC Genomics. 2011 Aug 24;12:426. doi: 10.1186/1471-2164-12-426 (PMC3218224; doi:10.1186/1471-2164-12-426)
Supplement: Additional file 5 — Schematic diagram of vitamin B6 metabolic pathway. The original vitamin B6 metabolic pathway diagram (collected from KEGG database) was modified by indicating DEGs and by showing their expression levels, after SPS1 was knocked down. [file 1471-2164-12-426-S5.PDF]

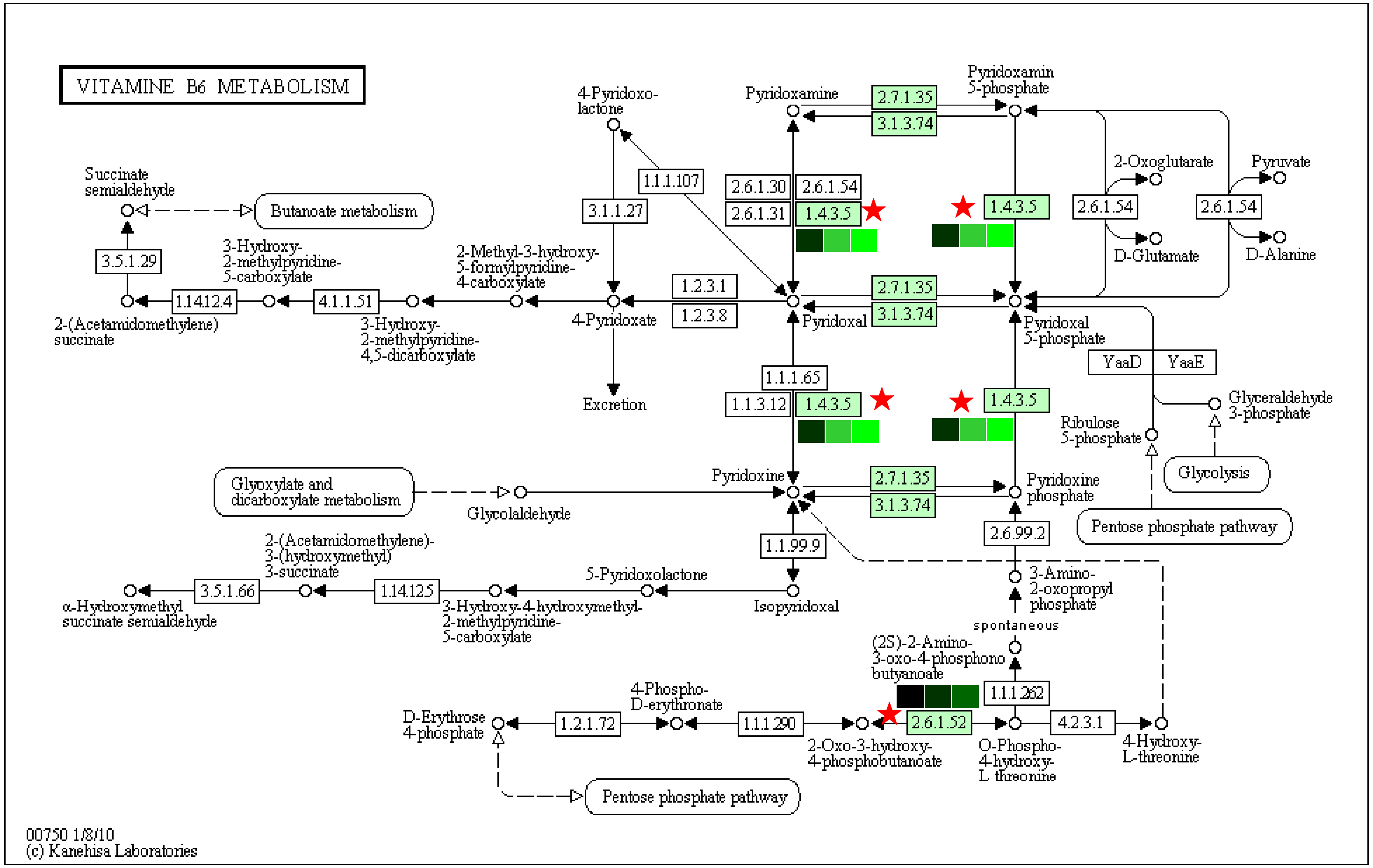

CG31472 (EC : 1.4.3.5) pyridoxine 5'-phosphate oxidase
CG11899 (EC : 2.6.1.52) phosphoserine aminotransferase
CG34455 (EC : 2.7.1.35) pyridoxine kinase
CG12237 (EC : 3.1.3.74) pyridoxal phosphaste phosphatase

Additional File 5. Schematic diagram of vitamin B6 metabolic pathway

Among four genes involved in vitamin B6 metabolic pathway in *Drosophila*, two genes were selected to DEGs (CG31472 and CG11899) and marked as red-colored star. Each green-colored boxes represents their temporal patterns in microarray (day 1, 3, 5). Reference map of vitamin B6 metabolic pathway was obtained from KEGG database (map00750).
