# Supplementary material for: Drosophila selenophosphate synthetase 1 regulates vitamin B6 metabolism: prediction and confirmation
Source: BMC Genomics. 2011 Aug 24;12:426. doi: 10.1186/1471-2164-12-426 (PMC3218224; doi:10.1186/1471-2164-12-426)
Supplement: Additional file 6 — Oligonucleotide sequences used as primers for RT-PCR or real-time PCR. List of all oligonucleotides used for RT-PCR and real-time PCR. [file 1471-2164-12-426-S6.PDF]

Additional File 6. Oligonucleotides sequences used as primers for RT-PCR or real-time PCR

Gene	Forward primer	Reverse primer
SPS1	tactaggccacgctcaaa	gccggttacaactgaatg
CG31472	gggattcaccttcttcacga	gtctgaccctgccagaactc
CG11899	tcccttcgatgtctccaagt	gccataccagcgactcctc
Egr	gcacataccggcaccacgcc	ttgcgatcgttgtgaatgtc
AttB	acaatctggatgccaaggtc	tacatctataccagggtaatat
AttD	agtttatggagcggtcaacg	aggtgatgattggcacttcc
CG8745	gcccagaacgaatttcttga	Aaattaggcggatcaacgtg
Cec B	aatccgatcgtaagccaaca	Agagaaatgagcgggtcgag
Drs	gtacttgttcgccctcttcg	acaggtctcgttgtcccaga
Dro	cataccgcggagaagtcatc	ttaggggacaaacccattca
Dpt B	atcctgatccccgagagatt	tgaagtgccctaaaacctgaa
PGRP-SD	atgacttggatcggtttgct	cccgttcttcgaagttacca
Mtk	ccaccgagctaagatgcaa	tgttaacgacatcagcagtgtg
W	gagaacgacaaaaggcgaag	actttctgctttcgctcctg
Oat	actcgtctatgccagggaga	gatgatctttgcctggttgg
GS1	gatcgcgttttggacaaagt	gacgtccgtccacgtctaat
Arg	cccaaggatcagctggttta	gagtgcctccacgatgct
Cyp6a8	ttccagagtcccgctgca	ccatgtctcttgtcaacc
PGRP-LF	ttacccgaccctattggttg	ttcattccccttgctttcag
Toll-7	gtctgcagcacagagacctg	gagcggcgaattctatgaac
RP49	cagtcggatcgatatgcta	aatctccttgcgcttctt
